# Identification of therapeutic targets for osteosarcoma by integrating single-cell RNA sequencing and network pharmacology

**DOI:** 10.3389/fphar.2022.1098800

**Published:** 2023-01-06

**Authors:** Yan Wang, Di Qin, Yiyao Gao, Yunxin Zhang, Yao Liu, Lihong Huang

**Affiliations:** ^1^ Science Research Center, China-Japan Union Hospital of Jilin University, Changchun, China; ^2^ Department of Geriatrics, China-Japan Union Hospital of Jilin University, Changchun, China; ^3^ Department of Gastrointestinal Colorectal and Anal Surgery, China-Japan Union Hospital of Jilin University, Changchun, China

**Keywords:** osteosarcoma, single-cell RNA sequencing, network pharmacology, molecular docking, therapeutic target

## Abstract

**Background:** Osteosarcoma (OS) is a common primary tumor with extensive heterogeneity. In this study, we used single-cell RNA sequencing (scRNA-seq) and network pharmacology to analyze effective targets for Osteosarcoma treatment.

**Methods:** The cell heterogeneity of the Osteosarcoma single-cell dataset GSE162454 was analyzed using the Seurat package. The bulk-RNA transcriptome dataset GSE36001 was downloaded and analyzed using the CIBERSORT algorithm. The key targets for OS therapy were determined using Pearson’s correlation analysis. Gene Ontology (GO) and Kyoto Encyclopedia of Genes and Genomes (KEGG) analyses were performed on key targets. The DeepDR algorithm was used to predict potential drugs for Osteosarcoma treatment. Molecular docking analysis was performed to verify the binding abilities of the predicted drugs and key targets. qRT-PCR assay was used to detect the expression of key targets in osteoblasts and OS cells.

**Results:** A total of 21 cell clusters were obtained based on the GSE162454 dataset, which were labeled as eight cell types by marker gene tagging. Four cell types (B cells, cancer-associated fibroblasts (CAFs), endothelial cells, and plasmocytes) were identified in Osteosarcoma and normal tissues, based on differences in cell abundance. In total, 17 key targets were identified by Pearson’s correlation analysis. GO and KEGG analysis showed that these 17 genes were associated with immune regulation pathways. Molecular docking analysis showed that RUNX2, OMD, and CD4 all bound well to vincristine, dexamethasone, and vinblastine. The expression of CD4, OMD, and JUN was decreased in Osteosarcoma cells compared with osteoblasts, whereas RUNX2 and COL9A3 expression was increased.

**Conclusion:** We identified five key targets (CD4, RUNX2, OMD, COL9A3, and JUN) that are associated with Osteosarcoma progression. Vincristine, dexamethasone, and vinblastine may form a promising drug–target pair with RUNX2, OMD, and CD4 for Osteosarcoma treatment.

## 1 Introduction

Osteosarcoma (OS) is a malignant tumor that occurs mostly in children and adolescents, and accounts for 20% of primary bone tumors worldwide (Zhu et al., 2019; Zhao et al., 2021). Currently, the methods of OS treatment mainly include surgery, chemotherapy, and radiotherapy (Rothzerg et al., 2022). However, current treatment methods for OS are unsatisfactory, with an overall 5 year survival rate of 65%–70% (Rothzerg et al., 2021). Therefore, there is an urgent need to develop novel treatment options for OS.

Gene mutations are considered the underlying cause of OS (Kuijjer et al., 2013). With the development of bioinformatics analyses, an increasing number of genes have been shown to be involved in the development of OS. For example, mutant p53 promotes cell proliferation, migration, and chemoresistance in OS (Tang et al., 2019a). LIM kinase one overexpression contributes to metastasis, invasion, and multidrug resistance in OS (Yang et al., 2018). Overexpression of Notch homolog protein three is associated with metastasis and poor prognosis in patients with OS (Tang et al., 2019b). All evidence suggests that bioinformatic analysis may provide valuable clues for the treatment of OS.

Single-cell sequencing (scRNA-seq) is a new bioinformatic analysis technique that fills the gap in other bioinformatic techniques for single-cell studies. Moreover, scRNA-seq technology has been prominent in exploring tumor heterogeneity and providing new therapeutic leads for the treatment of many cancers, including pancreatic cancer (Han et al., 2021), gastric cancer (Jiang et al., 2022), and Ewing sarcoma (Aynaud et al., 2020). Tumor heterogeneity plays an important role in cancer progression. Therefore, understanding the gene expression patterns of individual cells is particularly important in cancer treatment (Zhang et al., 2021a). OS exhibits great tumor heterogeneity; thus, we explored the potential targets for the clinical diagnosis and treatment of patients with OS using scRNA-seq and network pharmacology analysis.

## 2 Materials and methods

### 2.1 RNA sequencing (RNA-seq) data download and analysis

The single-cell dataset GSE162454 and bulk-RNA transcriptome dataset GSE36001 for OS were downloaded from the Gene Expression Omnibus (GEO, https://www.ncbi.nlm.nih.gov/gds) database.

Seurat 4.0 (Hao et al., 2021) was used for quality control, dimensionality reduction, clustering, and marker gene screening of the single-cell dataset, GSE162454. Cell types were annotated and differential gene analysis was performed using singleR (Aran et al., 2019). Subsequently, the CIBERSORT algorithm (Newman et al., 2015) was used to calculate the abundance of cell types in the OS and normal samples from GSE36001.

### 2.2 Identification of key targets in OS

OS-related targets were collected in the Genecards (Safran et al., 2010) and DisGeNET (Piñero et al., 2020) databases by searching with the keyword “*osteosarcoma*.” The search results were then merged and duplicates were deleted. Subsequently, these target genes were intersected with marker genes in the differentially expressed cells. Pearson correlation analysis was performed to screen genes whose expression levels were significantly correlated with differential cell type abundance (*p* <.05).

### 2.3 Protein-protein interaction network construction

The protein-protein interaction (PPI) network of key targets was analyzed using the STRING database (Szklarczyk et al., 2021) and visualized using the R packages ggraph (version 2.0.5) and igraph (version 1.3.1).

### 2.4 Functional enrichment analysis

Gene Ontology (GO) and Kyoto Encyclopedia of Genes and Genomes (KEGG) analyses of key targets were performed using the R package clusterProfiler (version 4.4.2) (Yu et al., 2012).

### 2.5 OS therapeutic drug prediction

In drug-disease association prediction, the deep learning-based algorithm deepDR (Zeng et al., 2019) learns the high-level features of drugs from a heterogeneous network through a multimodal deep autoencoder. Then, the learned low-dimensional representations of drugs and drug-disease pairs are encoded and decoded by the autoencoder to perform drug indication inference and filter the drug-disease pairs with high association based on the association score.

### 2.6 Drug–target interaction prediction

Drug-target interactions (DTI) are used to indicate the strength of the binding ability of a compound to a protein target. The deep-learning algorithm DeepPurpose (Huang et al., 2021) was used to perform DTI prediction using a simplified molecular-input line-entry system (SMILES) of compounds and amino acid sequence pairs of proteins as input data. Drug-target pairs with higher scores were screened based on the prediction scores.

### 2.7 Molecular docking analysis

The crystal structures of key target proteins were downloaded from the RCSB Protein Data Bank (http://www.pdb.org/) (Burley et al., 2017). Protein conformations were modified using PyMOL and AutoDock 1.5.6, including the removal of the original ligands and water, addition of hydrogen, optimization of amino acids, and calculation of charges (Seeliger and de Groot, 2010). The structure file of the drug in “mol2” format was downloaded *via* ZINC (https://zinc.docking.org/) (Sterling and Irwin, 2015). The downloaded protein and drug files were then converted to PDBQT format using Open Babel GUI software. Finally, molecular docking was performed using AutoDock 1.5.6, and the results were visualized using PyMOL. The screening criteria were binding energy less than −5.0 kcal/mol and the formation of hydrogen bonds between ligand receptors (Feng et al., 2021).

### 2.8 Cell culture

OS cell lines (U-2 OS, MG-63, and Saos-2) and human osteoblast cell line (hFOB1.19) were purchased from ATCC (VA, United States). OS cells were cultured in Dulbecco’s modified eagle medium (DMEM, Gibco, CA, United States) containing 10% fetal bovine serum (FBS) and 1% penicillin/streptomycin (Invitrogen, CA, United States). hFOB1.19 cells were maintained in DMEM/F-12 (Gibco) supplemented with 10% FBS, 2.5 mM L-glutamine, and .3 mg/mL geneticin (Invitrogen).

### 2.9 Quantitative real-time PCR (qRT-PCR)

Total RNAs were extracted with Trizol regent (Tiangen, China) and reverse-transcribed to cDNA using the Reverse Transcription Kit (Promega, China). Then, qRT-PCR was conducted on CFX96 Touch (Bio-Rad, Hercules, CA, United States) system with thermocycling conditions followed as 95°C for 3 min, 40 cycles for 95°C for 12 s and 62°C for 40 s. The relative expression of CD4, RUNX2, OMD, COL9A3, and JUN was calculated with 2^−ΔΔCT^ method and GAPDH as a housekeeping gene. The primer sequences of these genes are listed in Supplementary Table S1.

### 2.10 Statistics analysis

Data were expressed as mean ± standard deviation and analyzed by Prism 8.0 software with *t*-test or one-way ANOVA. *p* <.05 has a significant difference.

## 3 Results

### 3.1 Identification of cell clusters and dimension reduction analysis

**FIGURE 1:**
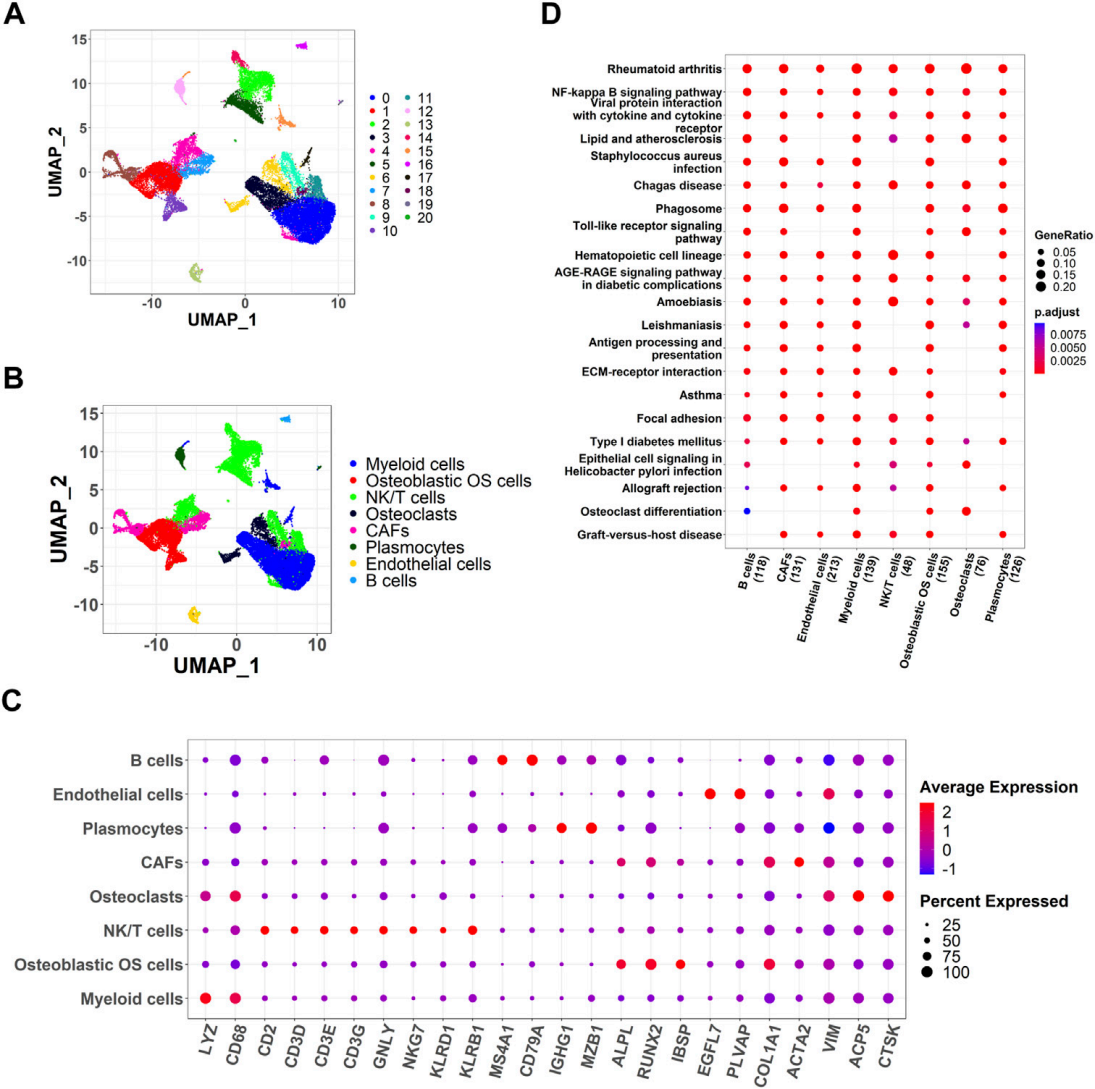
Identification of cell clusters and dimension reduction analysis. **(A)** Twenty-one cell clusters of dimension reduction analysis; **(B)** Ten cell types were identified by marker genes; **(C)** The expression of marker genes in eight cell types; **(D)** Kyoto Encyclopedia of Genes and Genomes (KEGG) enrichment analysis for marker genes of eight cell types.

Using scRNA-seq analysis, 21 cell clusters and eight cell types were identified (Figures 1A,B). Marker gene expression in the eight cell types was different between the different cell types (Figure 1C). KEGG enrichment analysis revealed significant heterogeneity in the enriched pathways for marker genes in the eight cell types (Figure 1D).

### 3.2 Collection of differential cell types

**FIGURE 2:**
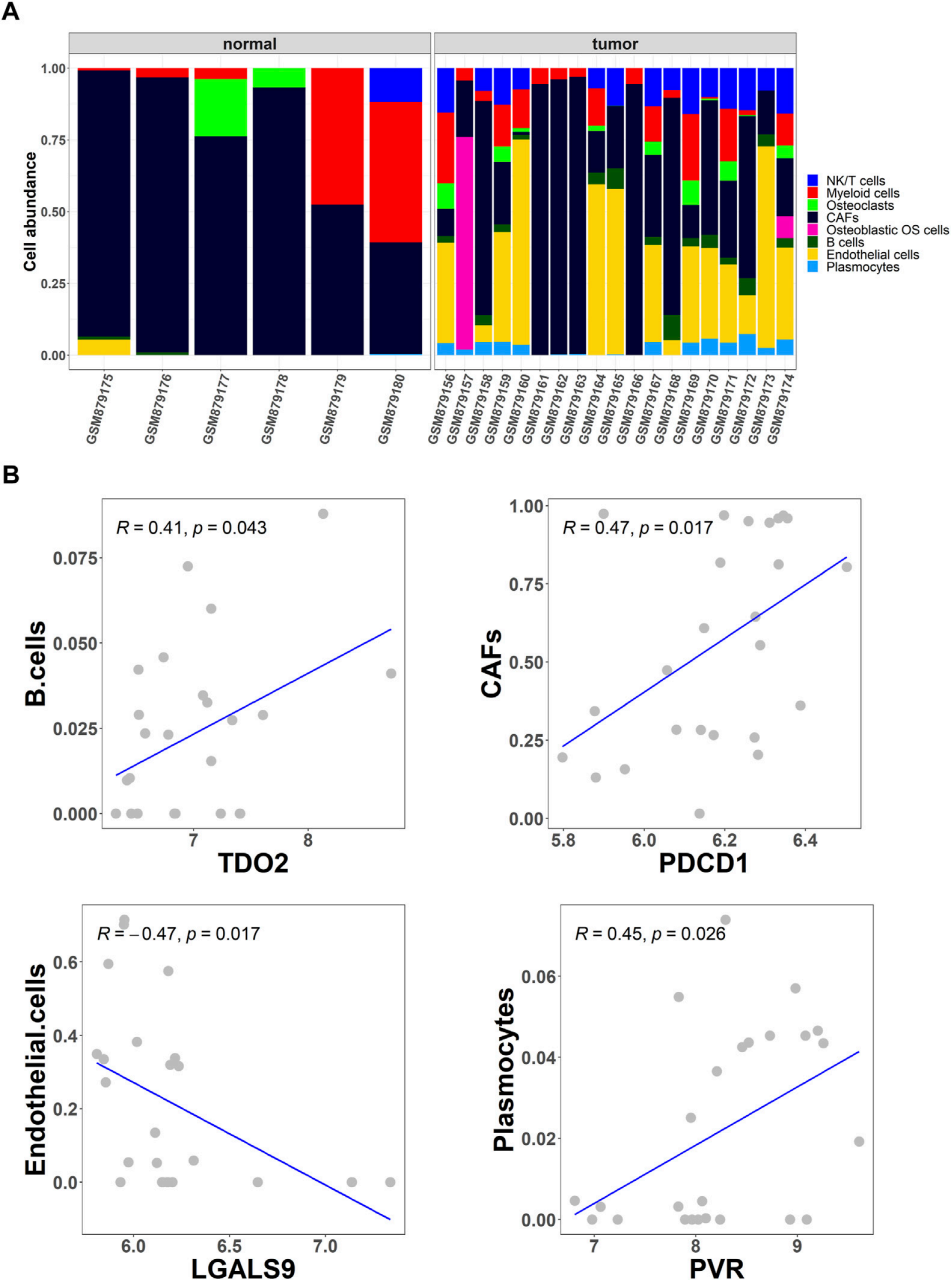
Collection of differential cell types. **(A)** The CIBERSORT algorithm was used to obtain the differential abundance of eight cell types in OS and normal tissues from the GSE36001 dataset; **(B)** Scatter plot of the correlation between the abundance of the four cell types (B cells, cancer-associated fibroblasts (CAFs), endothelial cells, and plasmocytes) and the expression of immune checkpoints TDO2, PDCD1, LGALS9, and PVR.

To further screen for differential cell types in OS, the abundance of eight cell types in OS and normal tissues from the GSE36001 dataset was analyzed using the CIBERSORT algorithm (Figure 2A). Studies show that immune checkpoints TDO2, PDCD1, LGALS9, and PVR play an important role in cancer treatment and prognosis (Stamm et al., 2018; Fan et al., 2020; Miao et al., 2020; Cui et al., 2022). Based on Pearson correlation analysis, cell abundance was screened to be significantly correlated with immune checkpoint expression levels, and the results showed that the abundance of B cells, cancer-associated fibroblasts (CAFs), endothelial cells, and plasmocytes were significantly correlated with immune checkpoints TDO2 (*p* = .043), PDCD1 (*p* = .017), LGALS9 (*p* = .017), and PVR (*p* = .026), respectively (Figure 2B).

### 3.3 Screening out key targets for OS

**FIGURE 3:**
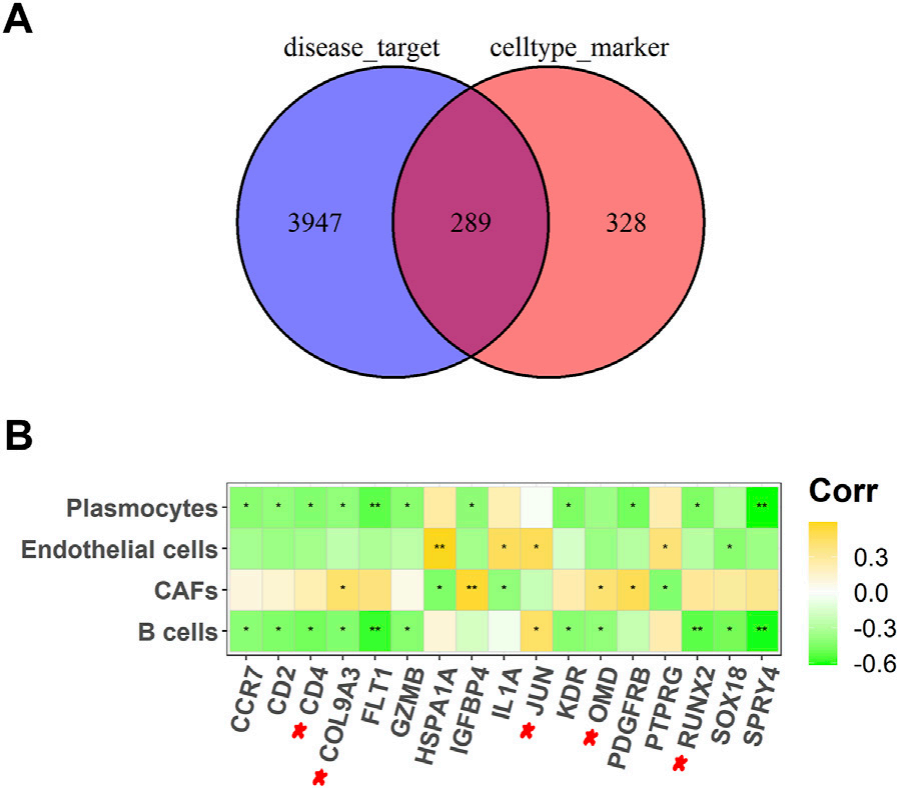
Screening out key targets for OS. **(A)** A Venn diagram of OS-related targets and four different cell types marker genes; **(B)** Heat map of 17 potential targets correlated with cell abundance in B cells, CAFs, endothelial cells, and plasmocytes. *p <.05, **p <.01.

A total of 4,236 OS-related targets were retrieved from the Genecards and DisGeNET databases. Next, overlapping with the marker genes of the four cell types, we obtained 289 common targets (Figure 3A). Finally, further screening by Pearson correlation analysis yielded 17 key targets (GZMB, IL1A, IGFBP4, JUN, KDR, CD2, FLT1, CCR7, CD4, COL9A3, SPRY4, OMD, RUNX2, PTPRG, HSPA1A, SOX18, and PDGFRB) that were significantly associated with the abundance of four differential cells (Figure 3B).

**FIGURE 4:**
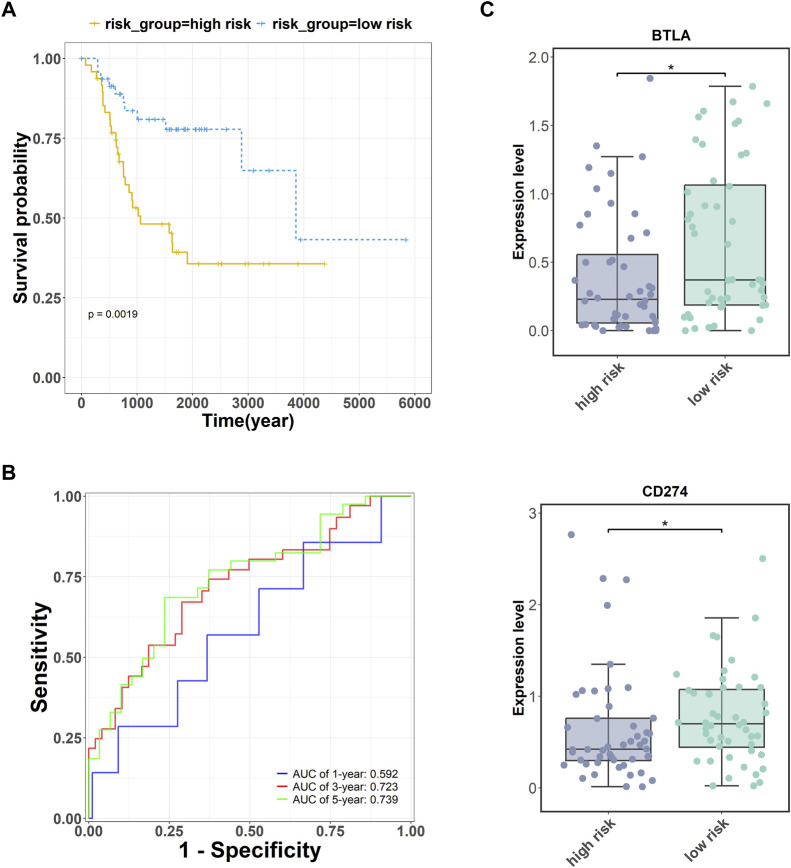
Prognostic analysis of five key targets (CD4, RUNX2, OMD, COL9A3, and JUN) in OS. **(A)** The survival analysis of these five genes. **(B)** The receiver operator characteristic (ROC) curve of these five genes. **(C)** The expression of immune checkpoints BTLA and PDL1 (CD274) were measured in the high- and low-risk groups. *p <.05.

Subsequently, we obtained expression data for 17 key targets corresponding to OS survival time information through the TARGET database (https://ocg.cancer.gov/programs/target). The five targets (CD4, RUNX2, OMD, COL9A3, JUN) may be the important prognostic factor *via* the multivariate Cox regression analysis. A combined prognostic marker model consisting of these five genes was constructed based on a multivariate Cox regression algorithm (Fisher and Lin, 1999). The risk calculation formula was as follows: Risk score=−0.31634×CD4+0.68318×RUNX2−0.26491×OMD+0.16464×COL9A3−0.25361×JUN . The results showed that the five key genes were able to accurately grade the risk of OS (*p = .0019*, Figure 4A). Moreover, the area under the curve (AUC) values of the receiver operator characteristic (ROC) curve were greater than .72 in the 3-, and 5-year survival analyses of OS (Figure 4B).

Additionally, cancer cells can undergo immune escape by dysregulating immune checkpoint proteins (Morad et al., 2021). To verify the accuracy of the high- and low-risk groupings of the five key genes, the expression of the immune checkpoints BTLA and PDL1 was detected. The expression levels of BTLA and PDL1 (CD274) were higher in the low-risk group than in the high-risk group (Figure 4C).

### 3.4 PPI network construction and functional enrichment analysis

**FIGURE 5:**
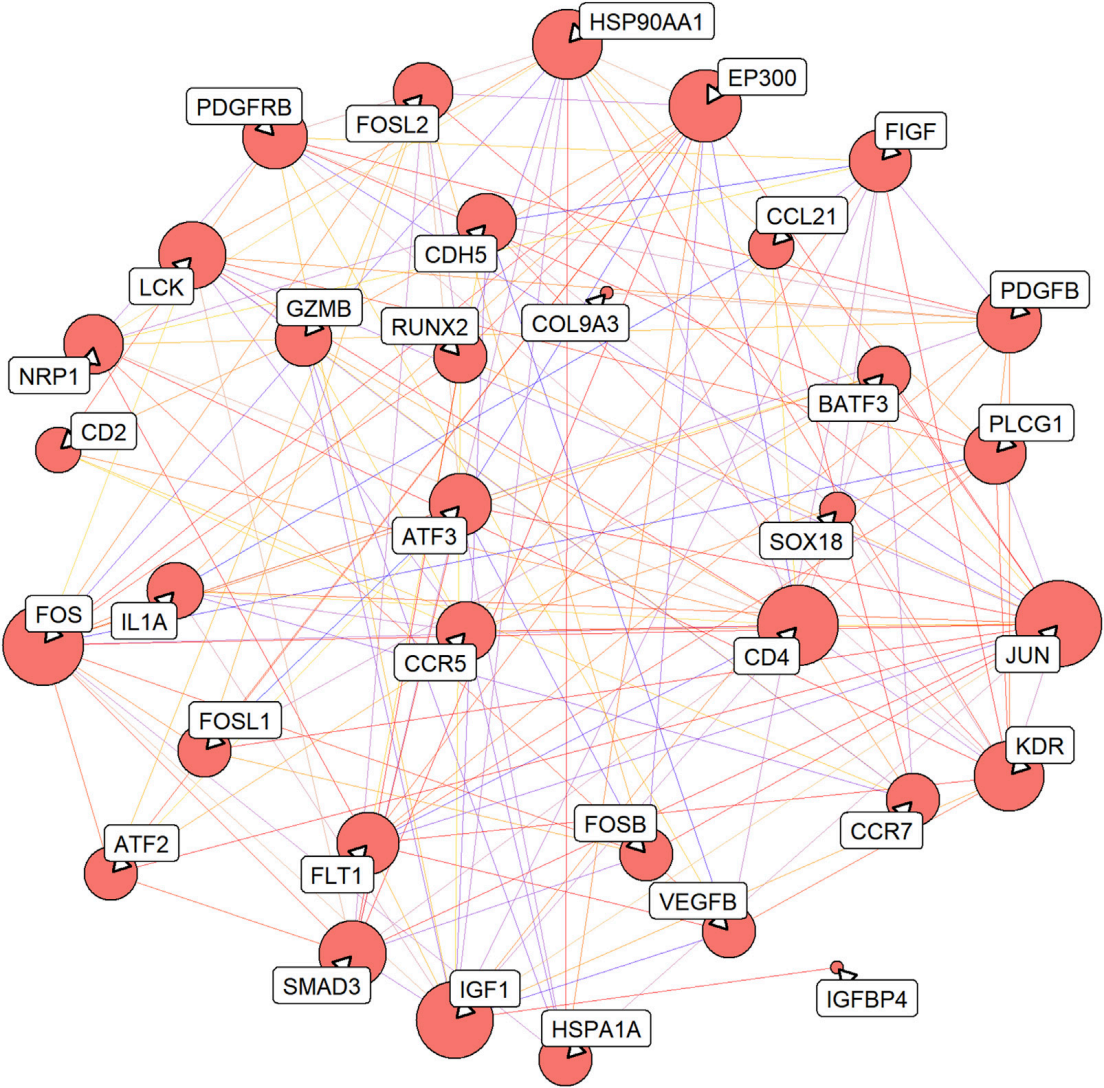
Protein-protein interaction (PPI) network of 17 key targets. The line from purple to red indicates a higher score of the reciprocal relationship, and the bigger size of the node indicates the higher degree.

**FIGURE 6:**
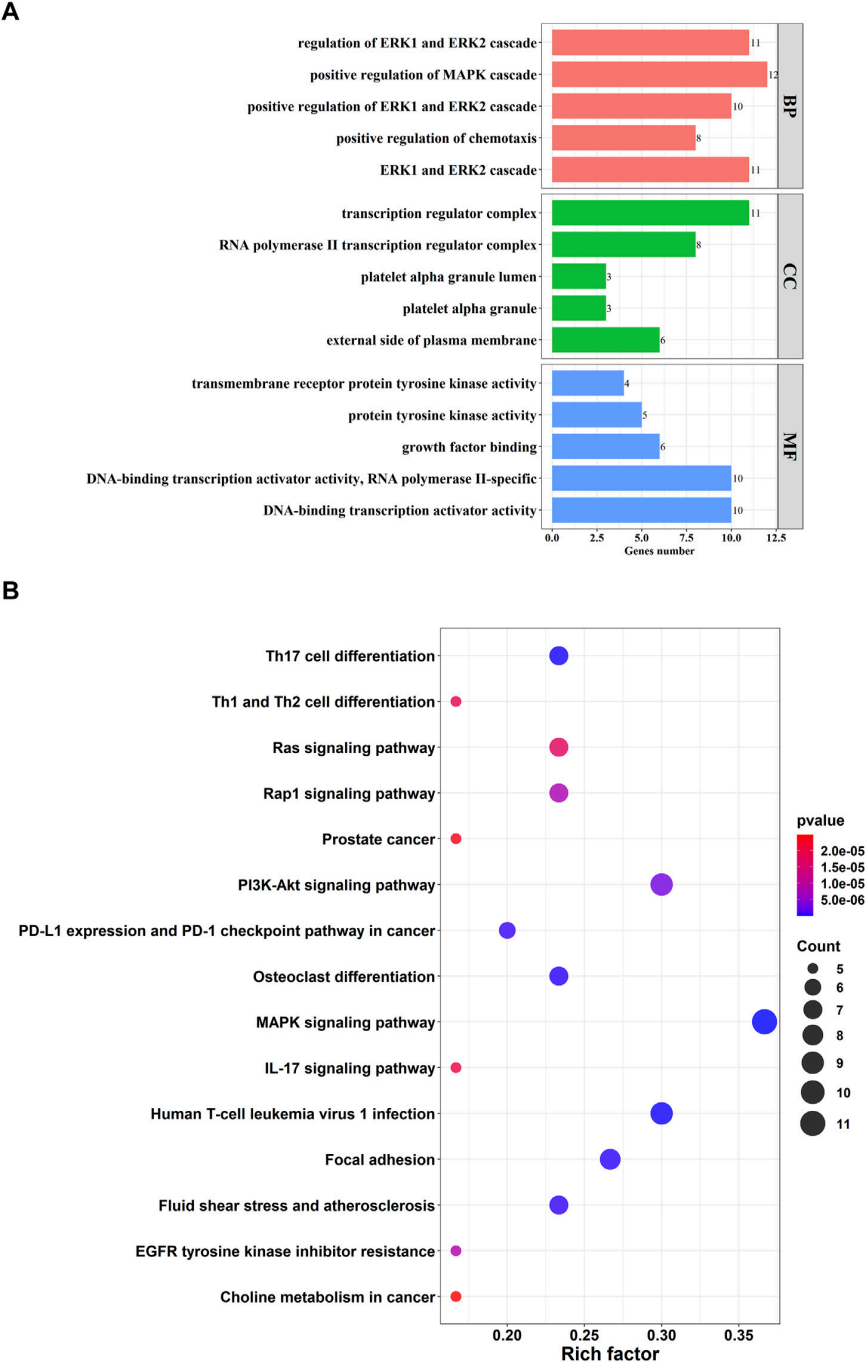
Function enrichment analysis of 17 key targets. **(A)** Top five terms of Gene Ontology (GO) enrichment analysis. GO consists of biological processes (BP), cellular component (CC), molecular function (MF); **(B)** Top 15 pathways of Kyoto Encyclopedia of Genes and Genomes (KEGG) enrichment analysis.

A total of 161 pairs of reciprocal relationships were obtained from the PPI network analysis of 17 key targets using the STRING database (Figure 5). GO and KEGG enrichment analyses were performed to explore the functions of the 17 key targets. GO enrichment analysis revealed 787 enriched terms (*p* <.05), and the top five terms of biological processes (BP), cellular component (CC), and molecular function (MF) are shown in Figure 6A. BP terms were primarily related to the regulation of ERK1 and ERK2 cascade, MAPK cascade, and chemotaxis. CC terms are located in the transcription regulator complex, RNA polymerase II transcription regulator complex, and external side of plasma membrane. The MF terms are associated with DNA-binding transcription activator activity, RNA polymerase II-specific, growth factor binding, and protein tyrosine kinase activity. Simultaneously, KEGG enrichment analysis was enriched in 77 pathways (*p* <.05), primarily in the MAPK signaling pathway, PI3K-Akt signaling pathway, and Human T-cell leukemia virus one infection (Figure 6B).

### 3.5 Drug–target interaction prediction

**FIGURE 7:**
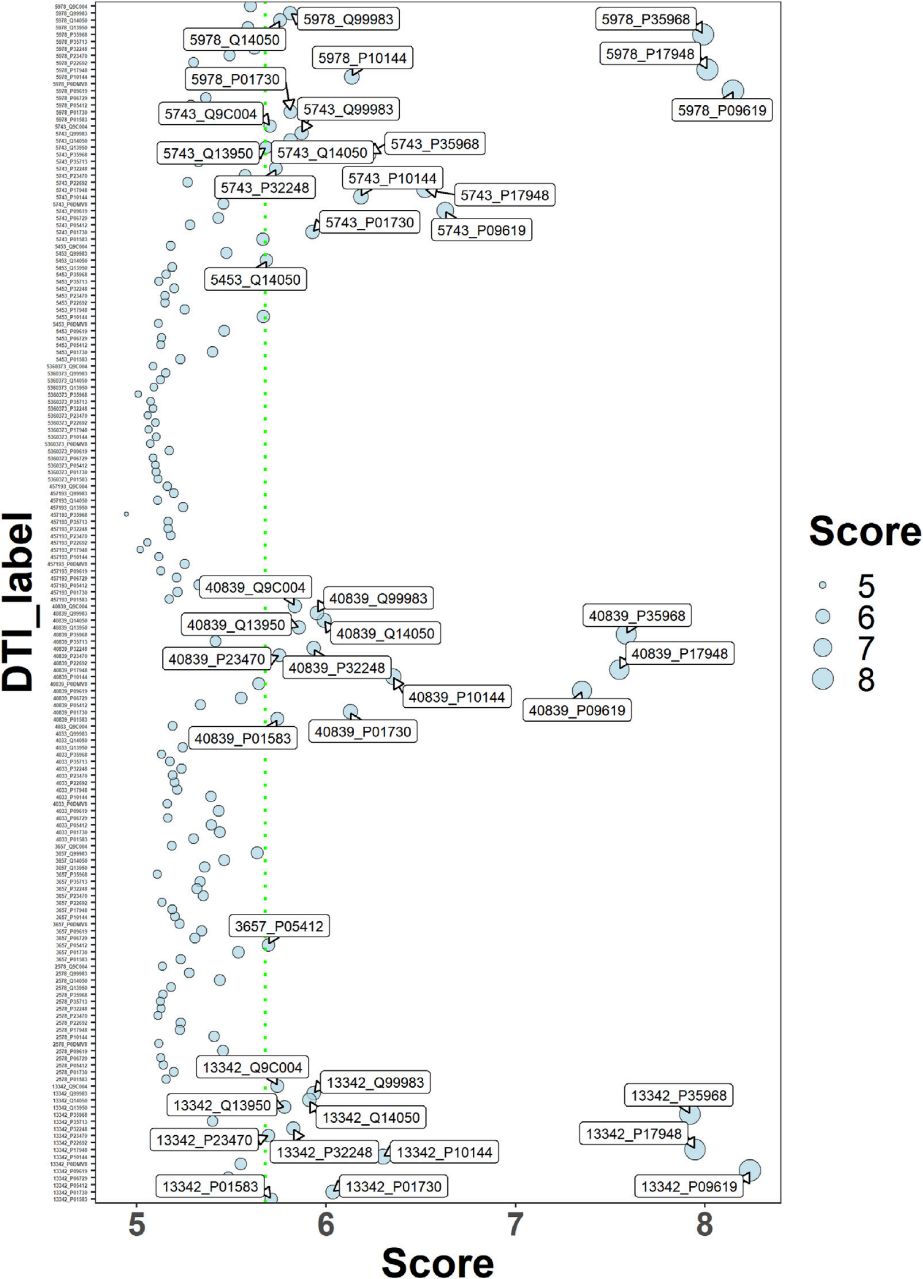
Drug-target interaction score dispersion points. The horizontal coordinate is the action score, and the vertical coordinate is the drug-target pair. The green dashed line is the 75% quartile of the score.

Using the deepDR algorithm, ten drugs with a high score association with OS were obtained (Table 1). The interaction relationship between 17 key targets and 10 drugs was predicted using the DeepPurpose algorithm (Figure 7). The results of screening scores with greater than 75% quartiles yielded six drugs (DB04572, DB01005, DB01234, DB00541, DB00570, and DB00309) that may act on 17 key targets (Table 2).

**TABLE 1:** The top 10 results of predict drugs against OS.

DrugID	DrugName	Pubchem_ID	DiseaseID	DiseaseName	predict.score
DB00541	Vincristine	5978	C0585442	Osteosarcoma of bone	.90791
DB00888	Mechlorethamine	4033	C0585442	Osteosarcoma of bone	.89119
DB00570	Vinblastine	13342	C0585442	Osteosarcoma of bone	.88823
DB04572	Thiotepa	5453	C0585442	Osteosarcoma of bone	.85945
DB00290	Bleomycin	5360373	C0585442	Osteosarcoma of bone	.83893
DB01234	Dexamethasone	5743	C0585442	Osteosarcoma of bone	.79852
DB00970	Dactinomycin	457193	C0585442	Osteosarcoma of bone	.78679
DB01005	Hydroxyurea	3657	C0585442	Osteosarcoma of bone	.76333
DB00309	Vindesine	40839	C0585442	Osteosarcoma of bone	.76033
DB00262	Carmustine	2578	C0585442	Osteosarcoma of bone	.75087

**TABLE 2:** Predicted drugs with interactions with 17 key targets.

Pubchem_ID	Drug_name	Target_uniprot	Target_gene	Score
40839	Vindesine	Q9C004	SPRY4	5.8344
13342	Vinblastine	Q9C004	SPRY4	5.7434
5743	Dexamethasone	Q9C004	SPRY4	5.7027
40839	Vindesine	Q13950	RUNX2	5.8562
13342	Vinblastine	Q13950	RUNX2	5.7793
5743	Dexamethasone	Q13950	RUNX2	5.6826
40839	Vindesine	P23470	PTPRG	5.7527
13342	Vinblastine	P23470	PTPRG	5.6951
40839	Vindesine	P09619	PDGFRB	7.3506
13342	Vinblastine	P09619	PDGFRB	8.2389
5978	Vincristine	P09619	PDGFRB	8.1492
5743	Dexamethasone	P09619	PDGFRB	6.6296
40839	Vindesine	Q99983	OMD	5.9503
13342	Vinblastine	Q99983	OMD	5.934
5978	Vincristine	Q99983	OMD	5.8099
5743	Dexamethasone	Q99983	OMD	5.8695
40839	Vindesine	P35968	KDR	7.5846
13342	Vinblastine	P35968	KDR	7.92
5978	Vincristine	P35968	KDR	7.9899
5743	Dexamethasone	P35968	KDR	6.2217
3657	Hydroxyurea	P05412	JUN	5.6947
40839	Vindesine	P01583	IL1A	5.742
13342	Vinblastine	P01583	IL1A	5.7095
40839	Vindesine	P10144	GZMB	6.353
13342	Vinblastine	P10144	GZMB	6.3015
5978	Vincristine	P10144	GZMB	6.135
5743	Dexamethasone	P10144	GZMB	6.1824
40839	Vindesine	P17948	FLT1	7.5469
13342	Vinblastine	P17948	FLT1	7.947
5978	Vincristine	P17948	FLT1	8.0129
5743	Dexamethasone	P17948	FLT1	6.5217
40839	Vindesine	Q14050	COL9A3	5.9884
13342	Vinblastine	Q14050	COL9A3	5.9115
5978	Vincristine	Q14050	COL9A3	5.7568
5743	Dexamethasone	Q14050	COL9A3	5.8115
5453	Thiotepa	Q14050	COL9A3	5.6851
40839	Vindesine	P01730	CD4	6.1276
13342	Vinblastine	P01730	CD4	6.0338
5978	Vincristine	P01730	CD4	5.8116
5743	Dexamethasone	P01730	CD4	5.9288
40839	Vindesine	P32248	CCR7	5.9338
13342	Vinblastine	P32248	CCR7	5.8254
5743	Dexamethasone	P32248	CCR7	5.7351

**FIGURE 8:**
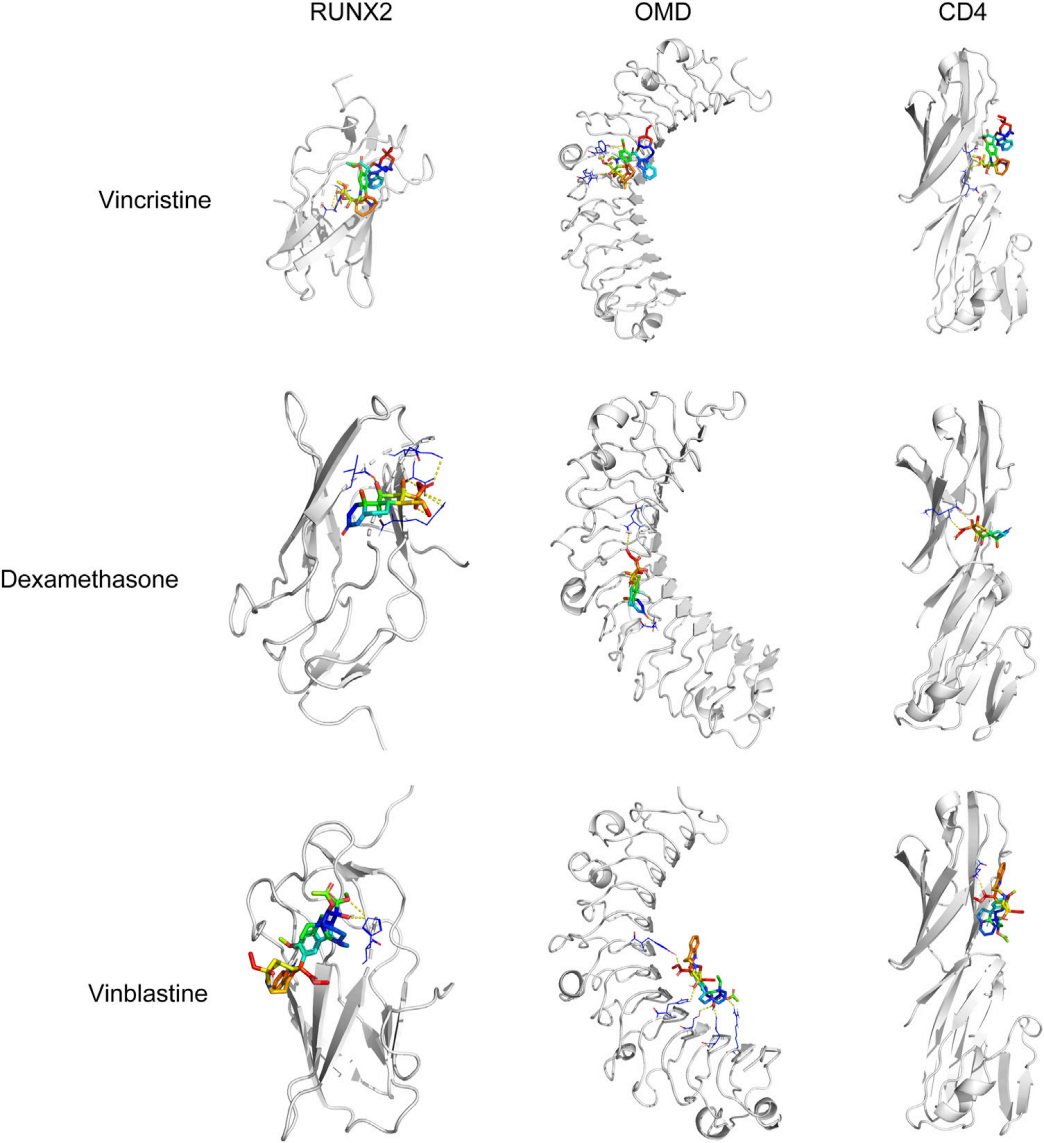
Molecular docking analysis of vincristine, dexamethasone, and vinblastine with RUNX2, OMD, and CD4.

To confirm that these six drugs were suitable for the treatment of OS, molecular docking analysis of six drugs and CD4, RUNX2, OMD, COL9A3, and JUN were performed using AutoDock Vina. The docking results showed RUNX2 has good binding affinity with vincristine (DB00541), vinblastine (DB00570), and dexamethasone (DB01234); OMD has good binding affinity with vincristine (DB00541), vinblastine (DB00570), and dexamethasone (DB01234); CD4 has good binding affinity with vincristine (DB00541), vinblastine (DB00570), and dexamethasone (DB01234) (Table 3; Figure 8).

**TABLE 3:** The information of molecular docking.

Drug_pubchem_id	DrugName	geneName	Uniprot	Free binding energy (kcal/mol)
5978	vincristine	RUNX2	Q13950	−5.1
13342	Vinblastine			−5.8
5743	Dexamethasone			−6.4
5978	vincristine	OMD	Q99983	−5.5
13342	Vinblastine			−5.6
5743	Dexamethasone			−6.9
5978	vincristine	CD4	P01730	−5.7
13342	Vinblastine			−6.1
5743	Dexamethasone			−6.3

### 3.6 Cell validation assay

**FIGURE 9:**
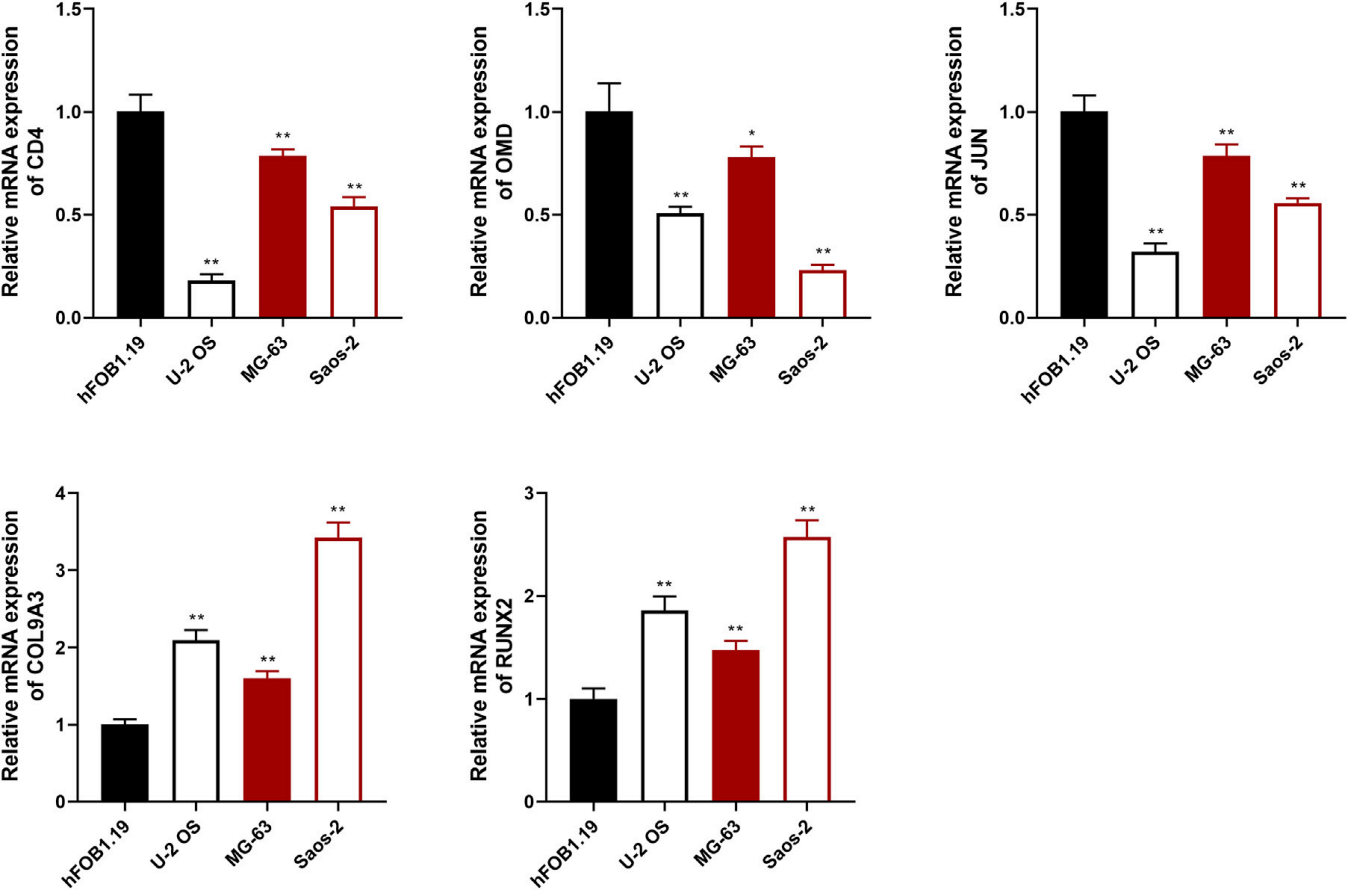
The relative expression of CD4, OMD, JUN, COL9A3, and RUNX2 was detected using qRT-PCR assay. *p <.05 and **p <.01 compared with the hFOB1.19 group.

To evaluate the prognostic effects of key targets on OS, qRT-PCR assay was used to detect the expression of CD4, RUNX2, OMD, COL9A3, and JUN in OS and osteoblast cells. The results showed that the expression of CD4, OMD, and JUN was decreased in OS cells compared with hFOB1.19 cells, whereas RUNX2 and COL9A3 expression was increased (*p* < .01; Figure 9).

## 4 Discussion

Over the past decades, traditional bioinformatics techniques have partially revealed the pathological mechanisms of OS; however, further research is still required. scRNA-seq technology can reveal the development of disease at the individual cell level. Therefore, in this study, we used scRNA-seq technology combined with network pharmacology analysis to explore effective therapeutic targets for OS treatment.

Tumors are heterogeneous cell populations that contain transformed cancer, supporting, and tumor-infiltrating cells (Prasetyanti and Medema, 2017). In this study, scRNA-seq with an unbiased approach was used to characterize cellular heterogeneity in OS. We identified the abundance of B cells, CAFs, endothelial cells, and plasmocytes has correction with the expression of immune checkpoints TDO2, PDCD1, LGALS9, and PVR. B cells are the main component of humoral immunity. It has been suggested that an increase in B cells is associated with good prognosis of OS (Li et al., 2021). CAFs are activated fibroblasts present within the tumor microenvironment that promote tumor cell growth, invasion, metastasis, and drug resistance (Zhang et al., 2021b). Based on these data, we confirmed that OS exhibits cell heterogeneity, and the differential abundance of cell types could affect the malignant progression of OS.

A total of 17 key targets were obtained from the intersection of marker genes of the four cell types and OS-related targets using Pearson correction analysis. Network pharmacology analysis was performed to determine the functions of the 17 key targets. GO analysis showed that these targets were involved in the regulation of ERK1 and ERK2 cascade, MAPK cascade, and chemotaxis. Reportedly, ERK signaling is involved in various cellular progress, such as proliferation, migration, and differentiation (Bonjardim, 2017). A study found that blocking ERK1/2 signaling pathway inhibits OS cell growth and metastasis (Yuan et al., 2020). Another study found that blocking the nuclear translocation of phosphor-ERK suppresses the migration and invasion of OS cells (Kim et al., 2021). MAPK signaling has a major effect on cell survival and apoptosis (Chen et al., 2016). It has been reported that activating the MAPK signaling pathway induces cell death in human OS (Lv et al., 2020). Zhou et al. (2020) found that inhibiting the function of TLR4-mediated MAPK-NF-ĸB signaling pathway against the oncogenesis of OS.

KEGG analysis showed that these targets act against OS *via* the MAPK signaling pathway, PI3K-Akt signaling pathway, human T-cell leukemia virus 1 infection, and so on. CD4-positive T cell are involved in the tumor immune environment in OS (Liu et al., 2020). T follicular helper (Tfh) cells are a subpopulation of CD4-positive T cell that may play an important role in the tumor microenvironment (Ochando and Braza, 2017). Study has shown that inhibition of the PI3K/Akt/mTOR pathway enhances the ability of OS Tfh cells to promote B cell maturation and immune function (Jiang et al., 2021). RUNX2 is an important transcription factor for bone development and osteoblast differentiation, and both metastasis and chemoresistance are associated with dysregulation of RUNX2 in OS (Vega et al., 2017). A review reported that mutual activation of the PI3K/Akt pathway and RUNX2 may be one of the main drivers of tumor progression or migration (Cohen-Solal et al., 2015). JUN is a factor of the JNK pathway, JNK pathway activation induces apoptosis and autophagy of OS cells (Li et al., 2020). In our study, qRT-PCR assay exhibited that the expression of CD4, OMD, and JUN was decreased in OS cells compared with hFOB1.19 cells, whereas RUNX2 and COL9A3 expression was increased. Moreover, multi-factor Cox regression algorithm and ROC curve analyses showed that five key genes are effective in the diagnosis and prognosis of OS. Furthermore, molecular docking results showed that vincristine, dexamethasone, and vinblastine all bound well to the key targets RUNX2, OMD, and CD4. Vincristine, dexamethasone, and vinblastine are common drugs used to treat OS (Boscoboinik et al., 1994; Ahlström et al., 2005; Chen et al., 2019). These results suggested that these five key targets could be potential targets for OS treatment.

In conclusion, four cell types (B cells, CAFs, endothelial cells, and plasmocytes) were identified in OS and normal tissues, based on differences in cell abundance. We identified five key targets (CD4, RUNX2, OMD, COL9A3, and JUN) that are associated with OS progression. GO and KEGG analysis showed that these five genes were associated with immune regulation pathways. Vincristine, dexamethasone, and vinblastine may form a promising drug–target pair with RUNX2, OMD, and CD4 for OS treatment. However, there are still shortcomings in our study. For example, we have only carried out a simple experimental validation of the bioinformatics results and our results lack the support of clinical results. Our study identified potential targets for the treatment of OS.

## Data Availability

The datasets presented in this study can be found in online repositories. The names of the repository/repositories and accession number(s) can be found in the article/Supplementary Material.
