# Supplementary material for: Identification of therapeutic targets for osteosarcoma by integrating single-cell RNA sequencing and network pharmacology
Source: Front Pharmacol. 2023 Jan 6;13:1098800. doi: 10.3389/fphar.2022.1098800 (PMC9853455; doi:10.3389/fphar.2022.1098800)
Supplement: Supplementary file 1 [file Table1.pdf]

Supplementary Material

Identification of therapeutic targets for osteosarcoma by integrating single-cell RNA sequencing and network pharmacology

Yan Wang, Di Qin, Yiyao Gao, Yunxin Zhang, Yao Liu, Lihong Huang*

*** Correspondence:** Lihong Huang: lhhuang@jlu.edu.cn

Table S1. The primer sequences for qRT-PCR assay.

Gene	Primer sequences (5'-3')	Length (bp)
CD4 forward	TGTACAGCTTCCCAGAAGAAGAG	264
CD4 reverse	GTTGGCAGTCAATCCGAACAC	
RUNX2 forward	TGGTTACTGTCATGGCGGGTA	101
RUNX2 reverse	TCTCAGATCGTTGAACCTTGCTA	
OMD forward	AGGCTGTGTCAGTGAATGCTT	82
OMD reverse	TGGGATAGTCTTGAGTTTGCGAT	
COL9A3 forward	GTGGATGGTCTGACTGGACG	287
COL9A3 reverse	GGGCAGATACTTGGGCACTG	
JUN forward	GCGGACCTTATGGCTACAGT	191
JUN reverse	CCCGTTGCTGGACTGGATTA	

GAPDH forward	GGAGCGAGATCCCTCCAAAAT	197
GAPDH reverse	GGCTGTTGTCATACTTCTCATGG	
